# The computational model lifecycle: Opportunities and challenges for computational medicine in the healthcare ecosystem

**DOI:** 10.1177/00368504251344145

**Published:** 2025-09-01

**Authors:** Sara Bridio, Pierre Deceuninck, Maurice Whelan, Andrew Worth

**Affiliations:** European Commission, Joint Research Centre, Ispra, Italy

**Keywords:** Computational medicine, in silico medicine, computer modelling and simulation, health data, model credibility, regulatory framework

## Abstract

Computational medicine promises significant advancements in healthcare, using physics-based simulations and artificial intelligence to optimise disease diagnosis, personalise treatment strategies and accelerate medical innovation. Biomedical research efforts are generating a growing number of computational models of human pathophysiology and medical treatments, with advanced applications in areas such as cardiovascular diseases, orthopaedics and cancer diagnosis. However, the widespread adoption of these models is hindered by technological and regulatory barriers. This article provides an overview of the potential impact, needs and challenges of the adoption of in silico medicine in the healthcare ecosystem, with a focus on initiatives to sustain this technology within the European Union's regulatory environment. The article introduces the concept of the ‘computational model lifecycle’ as a framework to describe the stages from academic research to pre-clinical and clinical applications, analysing key opportunities and challenges in translating these technologies at each stage. These challenges are associated with data management, standards for model credibility assessment, transparency of regulatory frameworks, and clinical integration. The article highlights European initiatives such as the European Health Data Space and the Virtual Human Twins Initiative, aimed at fostering the development and application of computational medicine in healthcare.

## Introduction

In recent years, computational (or in silico) medicine has become a prominent topic in biomedical research, with the development of increasingly sophisticated mathematical models and simulations of the human body, its physiology, pathology and treatment. Computational tools are being developed to support several stages of the patient healthcare pathway, including pathology diagnosis, pathology evolution, and (personalised) choice of treatment. Moreover, modelling and simulation is increasingly being used by industry in the development of new drugs and medical devices, and a considerable research effort is now focused on the development of the in silico trial (IST) technology, to assess their safety and efficacy with computational tools.

Computational models can be developed using a physics-based approach, when the mechanistic cause–effect relationships underlying the phenomenon are known. Typical examples of physics-based approaches used in biomedical research are finite-element (FE) structural mechanics, computational fluid dynamics (CFD) and physiologically-based pharmacokinetic/pharmacodynamics (PBPK/PD) modelling. In silico models can also be built using a data-driven approach, that is by using statistical or more sophisticated artificial intelligence (AI) techniques to identify input/output relationships from a large dataset of observations. Often, the two approaches are integrated, generating models that combine the solution of physics equations with data-driven information. Moreover, depending on its intended application, an in silico model could be the representation of a single cell, tissue or organ, or could be a multi-scale model, including all these elements at different spatial and temporal scales.

Advancements in computational medicine offer numerous potential advantages. For example, the digital twin technology promises to improve patient health, through faster and more accurate disease diagnosis and treatment personalisation. Moreover, in the context of medical product development, the use of in silico models enables a reduction in the costs and time associated with the use of in vitro tests, animal tests and clinical trials. From an ethical perspective, in silico approaches are a powerful means of fulfilling the 3Rs principles (Replacement, Reduction, and Refinement of animal use in research and testing), which is enshrined in the European Union (EU) legislation^1^ and is at the core of the activities of the European Union Reference Laboratory for Alternatives to Animal Testing (EURL ECVAM^2^). However, this potential is not fully exploited yet, as most in silico models are still limited to academic research.

**Figure 1.**
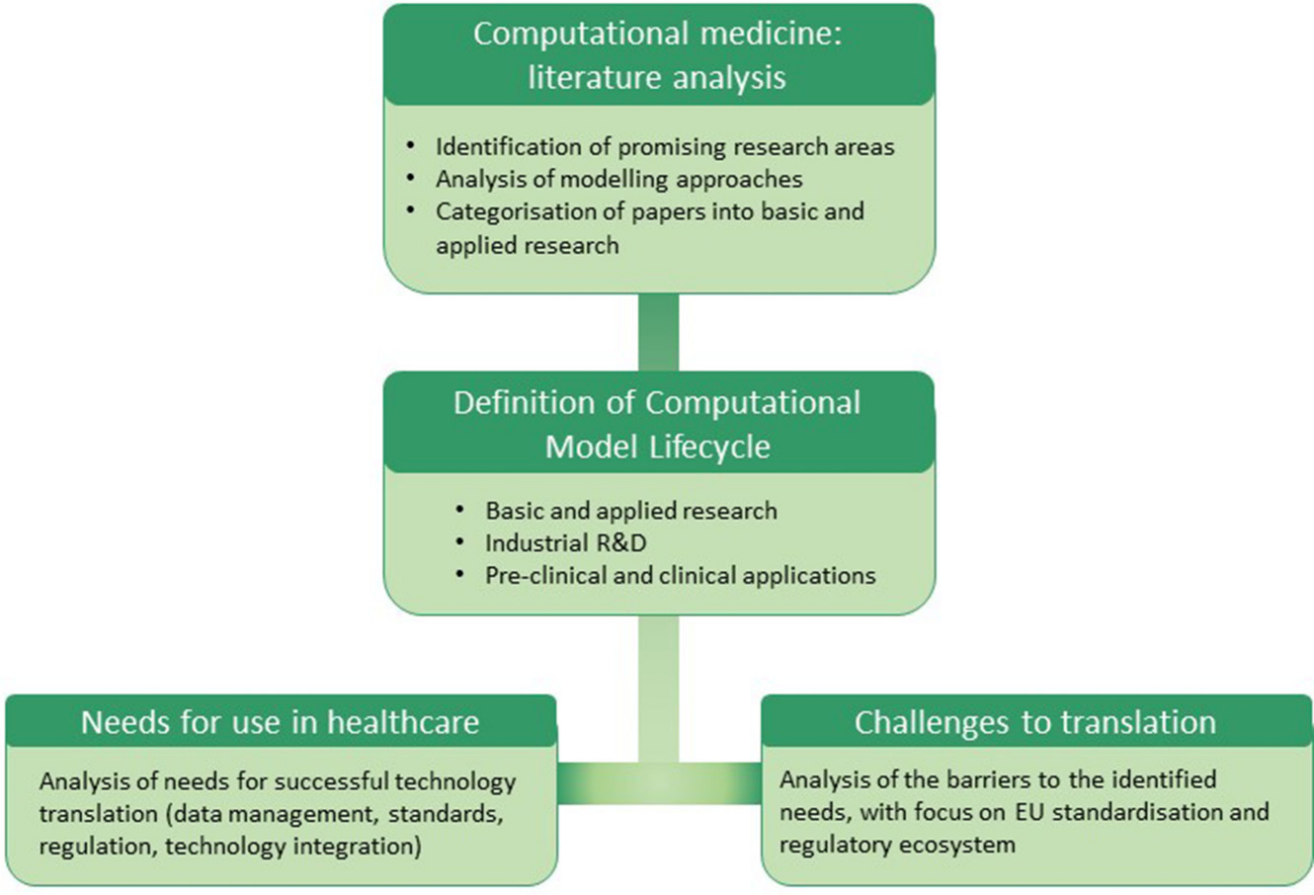
Flowchart of the literature review and analysis.

As summarised in the flowchart in Figure 1, after providing an overview of the most promising areas of application of different types of models in computational medicine, this paper introduces the concept of the ‘computational model lifecycle’, from academic research, to industrial research and development (R&D), up to pre-clinical and clinical applications. The lifecycle representation is used as a framework to analyse the main possible impacts of computational modelling in healthcare, as well as the needs and challenges for a faster adoption of these tools in pre-clinical and clinical settings. The analysis of barriers to the translation of in silico medicine technologies is focused on the EU's regulatory and qualification framework, highlighting gaps and initiatives aimed at enhancing the development and adoption of these technologies to improve the EU healthcare ecosystem.

## Computational medicine: Promising areas of application and modelling approaches

Computational medicine is being investigated in many areas of biomedical research. This section provides illustrative examples of how computational modelling is currently being applied in promising research areas. For technical details on the modelling approaches, we refer the reader to the relevant systematic reviews and research papers, cited below.

Among the applications, the one where computational medicine currently appears most mature, with sophisticated models being published in the literature and some clinical translation, is the treatment of cardiovascular pathologies. This is confirmed by the EURL ECVAM review on the use of non-animal models in biomedical research on cardiovascular diseases.^3^ Structural FE simulations^4^ and CFD^5^ are widely used to model patient-specific occluded coronary arteries, to understand which conditions favour the creation of atherosclerotic plaques, and to evaluate the best treatment option by simulating a balloon angioplasty or a stent implantation. High-fidelity modelling is also used to simulate the treatments for heart valve diseases. For example, transcatheter aortic valve implantation (TAVI) has been modelled in the literature with structural FE analyses.^6,7^ The resulting hemodynamics has been evaluated with CFD simulations,^8,9^ and fluid–structure interaction (FSI) approaches have been employed to model the mutual interaction between the blood flow and the implanted device.^10,11^ Similarly, computational models have been proposed in the literature for the treatment of thoracic or abdominal aortic aneurisms and dissections, which simulate patient-specific stent-graft implantations with an FE approach^12–15^ or FSI.^16–18^

Another field with an extensive computational modelling literature is the electrophysiology of the heart, where the aim is to improve treatments for cardiac arrhythmias. Mechanistic models of a single cardiomyocyte, the cardiac tissue or the three-dimensional (3D) heart provide insights into the pathophysiology of arrhythmias, and have been proposed as tools for treatment personalisation and drug development.^19–21^ Machine learning (ML) approaches have also been developed and are being explored for their potential to identify unknown relations between patient-specific characteristics and treatment outcomes.^21–23^

While most of the proposed in silico methodologies are still at an academic research level, some computational models in the cardiovascular field have received the approval of the Food and Drug Administration (FDA) and the EU's European Conformity (CE) marking, and are now used in clinical practice.^24,25^ The first application approved by the FDA as a software-based medical device is HeartFlow,^26^ which uses a computational model to personalise the treatment of coronary artery disease based on a patient's computed tomography (CT) scans. Similarly, FEops HEARTguide^27^ is a CE-marked and FDA approved^28^ software-based medical device, which supports the pre-operative planning of percutaneous procedures, such as TAVI and Left Atrial Appendage Occlusions (LAAO), through a patient-specific FE simulation of the clinical intervention. Among the first FDA approved solutions is the Medtronic CardioInsight Cardiac Mapping system,^29^ which combines the patient's electrocardiogram and CT scan to produce a map of the cardiac electrical activity and plan the treatment of arrhythmias. Another is SurgicalPreview,^30^ a computational platform that uses CT scans of a patient with a cerebrovascular aneurism to simulate the implantation of neurovascular devices and evaluate the treatment options. These examples, along with other solutions developed and approved over the years, show the utility of computational medicine tools in cardiovascular applications.

Computational models are being developed in numerous other areas of biomedical research. For example, in the orthopaedics field, mechanistic models are widely used to study the biomechanics of body joints in healthy and pathological conditions and to predict the possible outcomes of surgical interventions. Two types of mechanistic models are mainly used: multibody models, where the body segments of interest are modelled as rigid bodies and muscle and joint forces are estimated during different types of motion, and FE models, where accurate 3D representations of the joints are discretized to estimate stresses and strains exchanged between body segments and implanted prostheses.^31^ Examples can be found in the literature for various body joints and pathologies, including the treatment of osteoarthritis in the knee joint^32^ and the surgical correction of the scoliotic spine.^33^ Data-driven ML models are also emerging as tools to support the diagnosis of pathologies, to perform fracture risk analyses and to facilitate the personalisation of physics-based models.^34,35^

Finally, computational modelling has the potential to advance research on cancer treatment.^36^ AI technology is widely deployed to support the detection of tumour masses from clinical images, leading to earlier and more accurate diagnosis and classification.^37–39^ Moreover, AI techniques are used to evaluate the efficacy of pharmacological treatments, with a specific drug or combined therapies,^40–43^ enabling the repurposing of existing drugs.^44,45^ Agent-based modelling, which simulates the actions and interactions of autonomous agents to understand the behaviour of a (biological) system, has been widely used to simulate cancer growth and to study the effects of pharmacological and cell therapies,^46–51^ paving the way for research on innovative cancer treatments.

This overview clearly highlights a lively research interest in the development of in silico models of human pathologies and their treatment, with the ultimate goal of addressing important clinical needs. However, the potential applications of in silico tools at various stages of the healthcare pathway are still underexploited, as relatively few solutions have currently found an application outside academic research. In the next section, we propose the ‘computational model lifecycle’ as a means of breaking down the different stages of development and use of in silico medicine models. For each stage, we analyse the goals and possible impacts of modelling, and the translational challenges in research and healthcare.

## The ‘computational model lifecycle’

**Figure 2.**
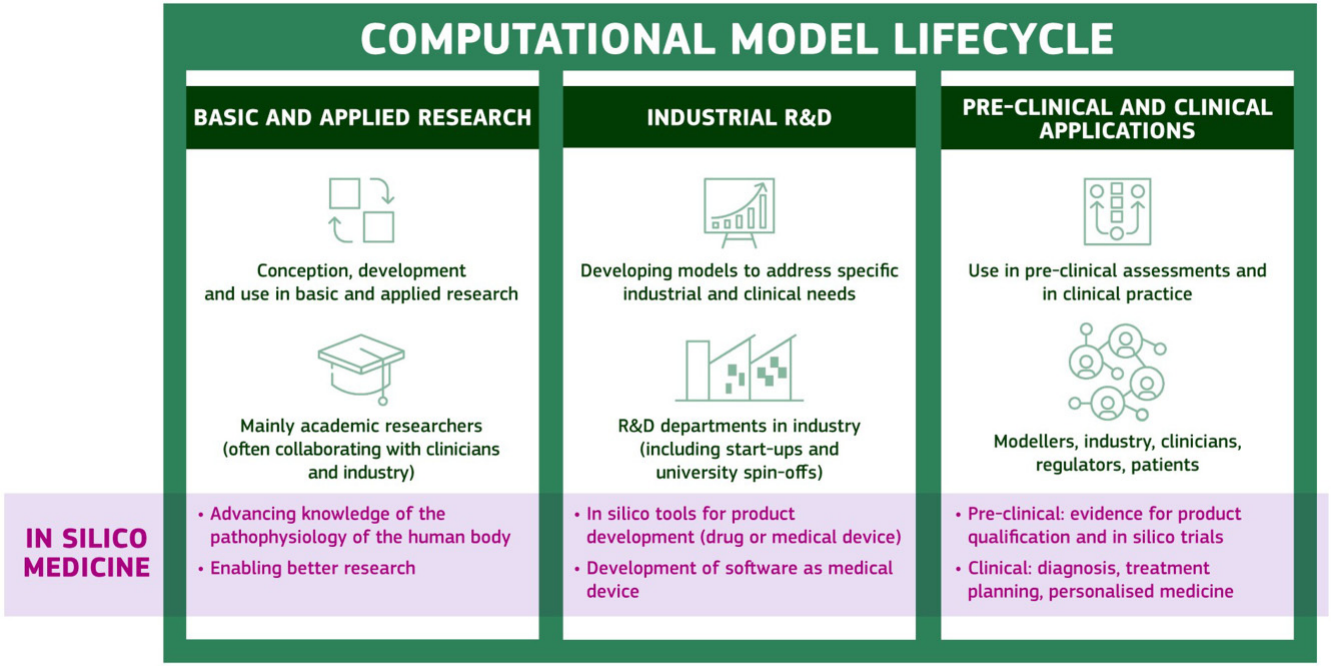
Representation of the computational model lifecycle.

We propose the ‘computational model lifecycle’ as a framework to describe the stages of development and translation of an in silico model in research and healthcare (Figure 2). By providing a representation of the journey of a computational model from its conception in academic research to its application in clinical settings, the lifecycle framework allows for a thorough analysis of the needs, challenges and impact at each stage, with the aim of highlighting the importance of transitioning models from research to practical, real-world healthcare settings. The framework defines distinct stages of model maturity, acknowledging the different levels of validation, regulatory compliance and ethical standards that models undergo throughout the lifecycle. This allows identification of bottlenecks or areas where the transition from one stage to the next is challenging, such as technical limitations or regulatory gaps, which is crucial for addressing them and facilitating a smoother progression of computational models into clinical practice.

The first stage of the lifecycle of a computational model is its conception, development and use in basic and applied research. This stage is predominantly driven by academic researchers, who frequently collaborate with clinicians and industry partners to identify key challenges that can be addressed through the development of computational solutions. The aim is to do better research. Indeed, the use of in silico models in biomedical research has demonstrated their potential for advancing knowledge of the pathophysiology of the human body. These new advanced approaches have the potential to overcome the limitations and costs of more traditional in vitro and in vivo experiments, which are often not representative of human physiology. In addition, the use of in silico technology has the advantage of reducing ethical concerns related to the use of animals in biomedical research.

For example, CFD investigations are being used to improve our knowledge on the causes of the formation of atherosclerotic plaques in coronary arteries,^52^ while FE modelling approaches help to understand the mechanisms of articular cartilage degeneration.^53^ The computational approach allows evaluation of mechanical quantities not easily measurable in vitro or in vivo, such as stress and strain distributions in tissues or devices. Multi-scale models combining different levels of detail from the organs to the tissues and single cells can be created, providing additional and more refined data for the investigation of pathophysiological mechanisms. In addition, emerging AI approaches are opening new opportunities for identifying cause–effect relations between patient characteristics and pathologies. In some of these cases, mechanistic models would be too complex to implement. For example, unsupervised ML techniques are being used for patient risk stratification, by performing biomarker clustering analyses to identify subgroups of patients with different prognostic predictions.^54–56^

When a computational model is considered sufficiently mature, and can address specific clinical and industrial needs, it can access the second step of the lifecycle, that is the R&D for commercial purposes. In this context, in silico models can be used for designing new medical devices or drugs, allowing the virtual testing of new solutions, and making the later stage of prototype testing more efficient.^57,58^ Alternatively, the product can be the model itself, commercialised as a software solution that enables clinicians to enhance disease diagnosis and inform personalised treatment planning (applications discussed below).

At this stage, the key players are industry-based researchers, frequently working in start-ups or university spin-offs. Their primary objective is to develop models that address specific industrial or clinical needs, tailored to a particular Context of Use (CoU). To ensure the reliability of these models, it is essential to rigorously assess the credibility of their predictions within the defined CoU. This validation process should be guided by established standards, where available. Notified bodies also play a crucial role at this stage, as they require specialised knowledge and expertise to evaluate the credibility of models that require certification, thereby ensuring compliance with regulatory requirements.

The final stage of the computational model lifecycle involves the deployment of in silico tools in pre-clinical and clinical settings, where they can be used to inform decision-making and drive improvements in patient care. This stage requires close collaboration among a diverse group of stakeholders, including modellers, industry partners, clinicians, regulators, and patients. By working together, these stakeholders can facilitate the effective translation of computational models into clinical practice, ultimately enhancing patient outcomes. Patients, in particular, have a critical role to play in this process, as they will be directly impacted by the use of the models. As such, it is essential that patients are fully informed about the potential benefits and limitations of in silico tools, and that their needs and concerns are taken into account throughout the deployment process.

Pre-clinical applications comprise the use of computational models to evaluate the safety and efficacy of medical devices and drugs. An emerging application is the implementation of ISTs, in which numerical models and simulations are used to assess the safety and efficacy of new treatments, devices or drugs, allowing the reduction, refinement and partial replacement of traditional clinical trials. A comprehensive analysis of the possible uses of ISTs is provided in Viceconti et al.^59^ For instance, ISTs can reduce the enrolment of real patients by augmenting the sample with the generation of virtual patients, or can enable the evaluation of a new treatment in under-represented patient phenotypes by defining virtual populations with the desired characteristics. Preliminary IST frameworks have been proposed in the literature, inter alia, for the development of tuberculosis^60^ and COVID-19 vaccines,^61^ for the evaluation of drugs in the treatment of cardiac arrhythmias,^62^ for assessing the efficacy of stroke treatments in different sub-populations and with different devices,^63^ and for the treatment of cerebral aneurisms with flow diverters.^64^

The clinical application of computational models offers a means of improving the management of patients’ diseases, through the implementation of new techniques for pathology diagnosis, and for the planning and personalisation of treatments.^65,66^ An example of computational modelling for disease diagnosis is the use of AI techniques for improving the identification of tumours from patients’ clinical images. This enables the early detection of tumours, improves the detection accuracy and supports the clinician in the final diagnosis.^38,39,67^ Similarly, ML approaches are being developed to support the early and accurate diagnosis of neurodegenerative diseases, such as Parkinson's and Alzheimer's diseases.^68,69^

In recent years, personalised medicine has become a major focus of research, with significant advances being made in the development of tailored treatments for various diseases. Several research initiatives are focused on the development of Virtual Human Twins (VHTs), that is patient-specific models of the body segment of interest (a 3D representation reconstructed from clinical images or a set of patient-specific data) used for simulating different treatment options. These models can support clinical decision-making, reduce the cost and duration of clinical procedures, and ultimately improve clinical outcomes. An interesting example is reported in Chatzizisis et al.,^70^ where the authors illustrate the first case of in silico pre-procedural planning for the treatment of left main coronary artery disease. Before surgical intervention, the patient-specific coronary anatomies of three patients were reconstructed from clinical images, and the obtained 3D domains were used to run computational simulations of the stent implantation, which allowed the clinical team to select the most appropriate treatment strategy.

In the orthopaedic field, the manufacturing of customised 3D-printed prostheses is emerging as a possible treatment option, which can be adapted to specific characteristics of the patient, optimising the clinical outcome. In this application, computational models play a crucial role in the design of the prosthesis and in the in silico testing (with FE simulations) of its mechanical properties and suitability for implantation.^71^ AI approaches are also being explored for treatment personalisation, for example in the treatment of spine pathologies,^72^ immune-mediated chronic inflammatory diseases^73^ and atrial fibrillation.^74^

It is therefore evident how computational medicine can improve healthcare management and patient outcomes by improving disease diagnosis and treatment personalisation. At the same time, these approaches contribute to a reduction in healthcare costs, both for hospitals and care centres, and for pharmaceutical and medical device companies. The use of in silico testing during product design and development allows the optimisation and de-risking of new medical products, identifying early in the process promising solutions and potential adverse effects.^57,58^ As discussed above, ISTs can help reduce the duration and the number of enrolled patients in clinical trials, reducing the cost and time-to-market associated with testing new treatments and devices.^75^ In clinical settings, computational medicine enables improved disease diagnosis, personalised treatments, optimisation of treatment protocols and predictive analyses of disease evolutions, all contributing to the more efficient management of patients, reducing the need of trial-and-error approaches and minimising ineffective treatments or costly complications.^68,70,71^

The following sections analyse needs and challenges to enable the translation of in silico models through the different stages of the computational model lifecycle, in order to fully leverage the potential impact of computational medicine in healthcare.

## Needs for the development and application of computational models in healthcare

**Figure 3.**
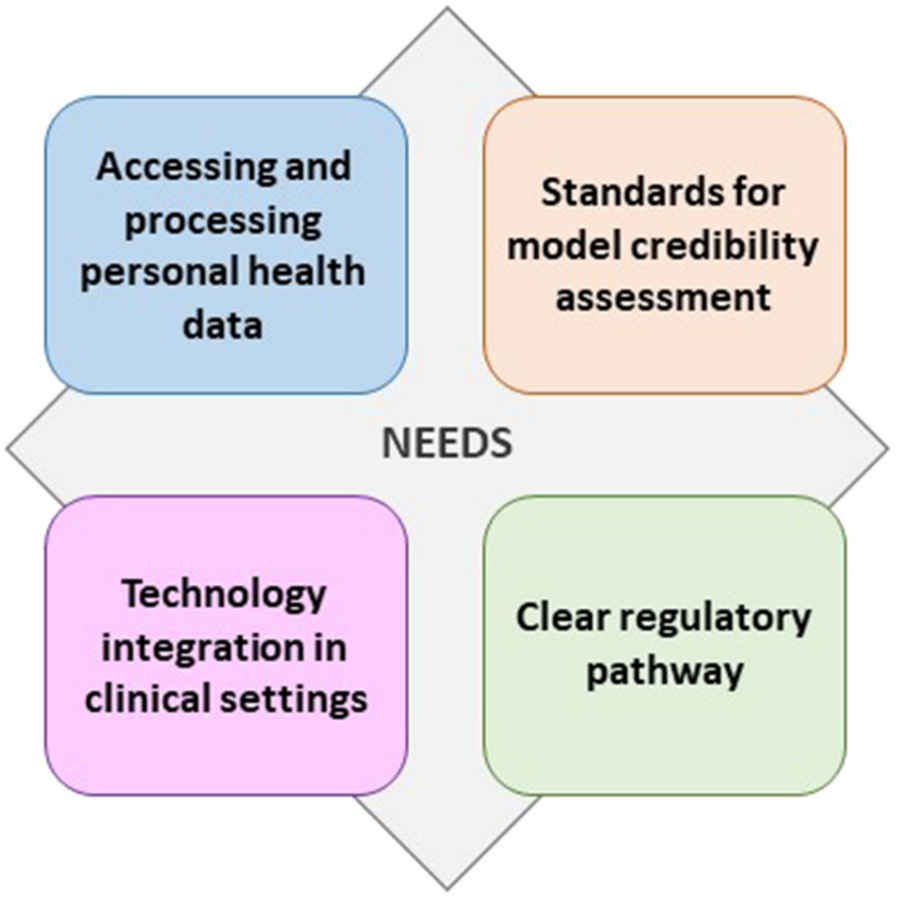
Needs for the successful translation of computational medicine in healthcare.

To proceed through the computational model lifecycle, key aspects related to model development and translation must be addressed, some relevant to the whole lifecycle, and others specific to a particular stage. The pre-requisites for the successful application of computational models can be divided in four main areas (Figure 3): data management, standards for model credibility assessment, regulation, and technology integration in the clinical setting.^76–80^

The management of data is of fundamental importance in all the stages of the model lifecycle, from the development of the in silico tool to the personalisation of patient treatment. First, data are needed for the development of in silico models, both for data-driven AI models and mechanistic simulations. Depending on the model type and its purpose, different kinds of human health data are needed, such as demographic and clinical history data (e.g. age, sex, weight, diabetes, hypertension), clinical images (e.g. CT scans, magnetic resonance imaging, ultrasounds), physiological signals (e.g. electrocardiograms, electroencephalograms), and genomic sequencing. Following model development, patient-specific data is required to validate its performance, ensuring that it can accurately reproduce expected outcomes when applied to individual patient characteristics, a crucial step in achieving personalised healthcare. Finally, to use the validated model in a real clinical scenario, the possibility of the clinical infrastructure to provide the needed patient's data must be guaranteed. Therefore, an adequate supply of human health data, both in terms of quantity (usually big in case of data-driven models) and quality, must be provided to model developers and made available to the final end-users of the in silico tool.

A key requirement, relevant to all lifecycle stages, but especially for commercialisation and clinical application, is the assessment of model credibility, that is the ability of the model to provide the expected output in the desired CoU. For personalised healthcare, a computational model must not only provide outputs within ranges observed in a certain human population, but it must be able to provide the correct prediction for the specific patient. The process of determining model credibility consists of three main steps: verification, validation and uncertainty quantification.^81^ The verification step ensures that the model is correctly built. For mechanistic models, this means checking that the governing equations are properly formulated and solved and, in case of FE, CFD or FSI analyses, that a proper sensitivity analysis is conducted on the spatial and temporal discretisation. For data-driven models, it means checking that the appropriate data pre-processing and learning algorithms are applied to the available dataset. The validation step consists of assessing to what extent the in silico model is able to reproduce the physical phenomenon it aims to replicate. Depending on the final purpose of the model, the validation step can be performed by replicating in vitro experiments, patient-specific clinical cases or the results of clinical trials. The acceptable accuracy of the model prediction must be defined based on the CoU of the model and the impact it may have on patient health. Finally, a process of uncertainty quantification must be performed, consisting of determining the variation of the output as a consequence of the uncertainties affecting the model, which are related to the modelling assumptions and the values of input parameters. For developers to conduct credibility assessments of their models, they require technical standards that outline a standardised, widely accepted methodology, ensuring consistency and rigour in the evaluation process.

In addition to establishing common standards for demonstrating model credibility, a clear regulatory pathway defined by certification authorities is needed to finally authorise the use of computational medicine technologies in pre-clinical assessments and in clinical practice. The presence of a well-defined qualification and regulatory framework will also promote the trust of healthcare professionals, patients and other stakeholders in the potential applications of computational approaches in healthcare management.

Finally, to enable the adoption of in silico tools, suitable technological platforms and devices must be integrated in the clinical setting. Such platforms can range from a simple software interface on a computer to more complex technologies allowing the acquisition of the needed data and the running of models and simulations. Collaboration between clinicians and modellers is essential and should be enhanced. On the one hand, clinicians should be more involved in the process of creating computational models to address clinical needs, mindful of the final users of the technology. On the other hand, clinical staff need to be trained in the use and interpretation of these technologies, or new figures should be recruited into healthcare teams with an expertise combining medical and computational knowledge.

## Challenges for fulfilling the identified needs with an EU focus

In this section, we discuss the barriers impeding the translation of in silico medicine tools, with a focus on the EU's standardisation and regulatory ecosystem, and on European initiatives promoting the development of the technology.

Most of the challenges in translating promising computational medicine models to applications in healthcare are related to a lack of standardisation and regulation for their development and adoption.

The use of personal health data for creating in silico models poses issues related to data availability, quality and sharing.^65,82,83^ To collect patient-specific data, model developers need to collaborate with clinicians, who can provide data after receiving the informed consent from the patients. A first issue is related to data anonymization and patient privacy protection, for which clearly defined standards and regulations are required. Moreover, a standardised approach is also required to ensure the collected data satisfy quality criteria, such as completeness, consistency, accuracy and precision, tailored to the purpose of their use. Together with data quality, data harmonisation and standardised frameworks for data sharing are important to allow the models to operate with data collected in different healthcare centres. Finally, with the growing adoption of AI approaches and interest in personalised medicine, the availability of large, complete and accessible human health datasets is becoming an important research need, due to the difficulties of data collection. Therefore, there is a growing demand for the creation of open-access health data repositories, which researchers can access and use for model development and validation.

To enhance the usability and sharing of scientific data, including health data, there is a wide consensus around the Findable, Accessible, Interoperable and Reusable principles.^84^ Adherence to these principles and common standards for data generation and sharing allow the full potential of data analysis and computational modelling to be leveraged in healthcare.^85^ Moreover, to make better use of already available datasets, there is a need for regulatory guidelines on the possible reuse of patient data for new research studies, beyond the ones they were originally collected for (the ‘secondary uses’ of health data). These guidelines could also partially address ethical concerns associated with the use of AI in healthcare. For example, a lack of transparency and explainability of the algorithms can erode the trust of patients and clinicians. In addition, the use of existing data can produce biased models, with the risk of perpetuating existing health disparities and the unequal treatment of underrepresented patient populations.^83,86^

The European Commission (EC) is developing a regulatory framework that can address these data management challenges. The rules for handling personal data in the EU, including health data, are defined in the General Data Protection Regulation (GDPR, Regulation (EU) 2016/679),^87^ in force since 2018. The GDPR regulates data privacy, anonymisation and pseudonymisation, the transparency on the use of data, and rules on data sharing. It also affirmed the possibility of a secondary use of health data for research purposes, but without detailed legislation, which was delegated to member states. More recently, as part of the EC's European Strategy for Data, the Data Governance Act (Regulation (EU) 2022/868),^88^ applicable since September 2023, has the aim of increasing trust in data sharing, promoting strategies to increase data availability and overcoming existing barriers to data reusability in different key sectors, including health care. Moreover, the EU recently adopted a regulation for the creation of the European Health Data Space (EHDS, Regulation (EU) 2025/327),^89^ a health-specific data sharing framework through which patients, doctors, researchers and policy makers can have a safe and regulated access to health data throughout the EU. The expected benefits of the EHDS are to empower citizens, providing them with greater control and seamless access to their electronic health data in any EU Member State (primary use of health data), and to enable the re-use of health data for research, innovation, policy-making and regulatory activities, in full compliance and complementing EU data protection regulations (secondary use of health data). The regulation defines security and interoperability criteria for systems that process electronic health data, ensuring data quality, consistency and reliability in data exchanges across EU borders.

Concerning data-driven modelling, the EU recently adopted the world's first regulation on AI technology. The Artificial Intelligence Act (Regulation (EU) 2024/1689)^90^ establishes different obligations based on the risks and impacts associated with AI systems. AI models used as medical devices (e.g. to support clinical decision-making) are classified as high-risk AI systems, with the need to satisfy mandatory requirements, such as an adequate risk assessment and mitigation plan, high quality of the training dataset, high level of accuracy and transparency towards users.^91^ The requirements, among others, to detect and correct data biases (Art.10), to ensure an appropriate degree of transparency (Art. 13), and to foresee a human oversight (Art. 14) are intended to address the ethical concerns related to the use of the AI technology in healthcare.

Clear standards and regulatory frameworks are also needed for the credibility assessment of computational models and their approval for use in healthcare.^82,92^ In the EU, there are different qualification and regulatory pathways depending on whether in silico technologies are applied to drugs or medical devices. The European Medicines Agency (EMA) offers scientific advice for the qualification of innovative development technologies, including computational modelling, for pharmaceuticals.^93^ It also provides guidelines on the reporting of computational PBPK model results in regulatory submissions, such as the authorisation of new drugs or clinical trials.^94^

A similar qualification pathway does not currently exist in the EU for medical device development methodologies. The EU Medical Devices Regulation (Regulation (EU) 2017/745^95^) recognises computational tools used for treatment or diagnosis as ‘Medical Device Software’ and requires a CE-marking procedure for these technologies. Moreover, it mentions ‘computer modelling’ among the testing methods for pre-clinical evaluation of medical devices, but there is no EU harmonised standard on how to produce evidence of the credibility of computational models predicting the safety and efficacy of new products.

A comprehensive analysis of the need to establish a ‘Good Simulation Practice’, that is a summary of best practices in computer modelling chosen as a standard by the modelling community, can be found in Viceconti and Emili.^80^ The document also provides a systematic review of the existing international standards and regulations related to the development, management and use of computational models. The review briefly mentions a Japanese guideline on the in silico evaluation of medical devices and a Chinese guideline for model-informed drug development. However, most of the available guidance documents are provided by EU or U.S. regulatory agencies.

In the United States, the FDA provides regulatory guidelines both for drugs and medical devices. For drug development and approval, the FDA has provided guidelines on the development, validation and use of Population Pharmacokinetics (Pop-PK),^96^ exposure-response relationships^97^ and PBPK models.^98^ For medical devices, the FDA published in 2016 the final version of a guideline on reporting the results of computational models in medical device regulatory submissions.^99^ Subsequently, the American Society of Mechanical Engineers (ASME) published in 2018 a technical standard entitled ‘Assessing Credibility of Computational Modelling through Verification and Validation: Application to Medical Devices’,^100^ referred to as ‘ASME VV40-2018’. This provides a framework to perform verification, validation and uncertainty quantification of computational models for medical devices. The framework proposes that the demonstrated level of credibility must be proportional to the risk associated to the use of the computational model, that is how much it is relied on in clinical decision-making or in the functioning of another medical device, and how severe the consequences of incorrect model predictions would be for patients. The ASME VV40-2018 standard was then adopted by the FDA as a valid framework for reporting computational evidence in medical device submissions. A document released by the FDA in November 2023 expands on the accepted sources of evidence for model validation, providing a more general framework with respect to the ASME VV40-2018 standard for demonstrating the credibility of computational models.^101^

A similar approach could be considered in the EU, establishing guidelines allowing a greater reliance on in silico evidence in regulatory submissions for medical devices. The ASME VV40-2018 refers to physics-based models, and does not include a framework for the credibility assessment of data-driven or hybrid models. Since these approaches are increasingly being integrated in the development of in silico tools for healthcare, an EU standard for model development and credibility assessment should consider both approaches. Both for physics-based and data-driven models, a wider and clearer EU regulatory framework is needed to take advantage of the opportunities offered by computational medicine for the pre-clinical testing of new medical devices and drugs, for healthcare personalisation and to facilitate the adoption of new treatments through ISTs.

To enable the use of in silico medicine in clinics, computational tools must be integrated effectively with healthcare systems, which poses several challenges.^83,102^ Computational medicine tools may use different data formats and standards, and different communication protocols, making it difficult to integrate with existing healthcare systems. Privacy protection and security in the exchange of patients’ data must be always guaranteed. Another challenge is the availability and scalability of computational resources. Data-driven models, once trained on large datasets, can usually be deployed as software that can easily run on common computers. However, if the models need to be retrained with new data from clinics, or if large amounts of patient data need to be analysed, powerful computing resources are needed, which are usually not available in clinical environments. Similarly, mechanistic models such as FEM, CFD and FSI are usually computationally demanding, requiring high-performance computers to run simulations of clinical procedures in patient-specific domains. Possible solutions would be to externalise the calculations or to use cloud computing platforms, posing security and legal issues related to the transfer of personal health data from the clinics to computing facilities. Additionally, computational medicine tools may not be designed to fit seamlessly into existing clinical workflows, requiring additional training and support for healthcare professionals.

In this context, FEops HEARTguide is a successful example of translation of a computational model from academic research to commercialisation and clinical application. FEops HEARTguide is a pre-operative planning tool that supports the physician in defining the best treatment for cardiac procedures such as TAVI and LAAO. This software-based medical device operates through a cloud-based platform, where the physician uploads CT images of the patient's heart anatomy. The confidentiality of the data is guaranteed in line with national privacy legislation (the European GDPR and the American Health Insurance Portability and Accountability Act).^27^ From these images, a 3D reconstruction of the patient-specific anatomy is obtained and FE simulations of the implantation of structural heart devices (prosthetic valve or left atrial appendage occluder) are conducted, predicting the outcome of procedures varying in device design, size and location. The 3D configurations of the simulated implantations are sent to the physician through the cloud platform, helping the selection of the optimal intervention for the specific patient. FEops HEARTguide is CE-marked and FDA-cleared. The documentation for the ‘De novo classification’ request to the FDA for the LAAO planning tool is publicly available and provides information about the process of credibility assessment of the computational model.^28^ After describing the model and its indication for use as a medical device, the document lists a summary of non-clinical studies demonstrating the reliability of the model. Software documentation and cybersecurity information were provided in accordance with FDA guidelines. Software verification, validation and credibility assessment were conducted following the ASME VV40-2018 standard: the rigour required for each assessment was determined by considering the model's CoU and the associated risk. A repeatability and reproducibility study was also provided. Finally, a validation exercise with clinical cases was conducted, to determine if the model accuracy was clinically acceptable for the defined CoU. This example shows how a set of well-defined guidelines and standards can help in building confidence in in silico technologies and in facilitating the translation to commercialisation and clinical use.

A study on clinicians’ awareness, trust and use of computational medicine tools^103^ was conducted between 2020 and 2021 by the Virtual Physiological Human (VPH) Institute,^104^ an international non-profit organisation whose mission is to promote the realisation and adoption of the VPH technology in biomedical research and in the clinics. A survey was conducted among the clinical community to understand their level of knowledge and acceptance of computer modelling and simulation, together with their reported experiences with associated barriers and perceived benefits. Results showed a good awareness of computational technologies, although the usage frequency is still low. Fields with the highest usage of in silico modelling are cardiovascular and musculoskeletal, and mostly for the planning of interventions. A perceived benefit is the increased trust in the planning of clinical procedures, and in general a good level of trust in the technology was found among responders. Among the barriers, access to computing resources and the time required to obtain results from simulations were the most reported. The study acknowledged some limitations due to the size of the interviewed sample, and a possible bias towards clinicians already aware of the technology. Nonetheless, the results appear encouraging for the willingness of healthcare providers to rely more on computational medicine.

A thorough analysis of the barriers preventing the adoption of the VHT technologies can be found in the ‘First draft of the VHT roadmap’, proposed by the EDITH consortium.^76^ EDITH^105^ is a Coordination and Support Action, funded by the EC, which aims at developing a roadmap towards the creation of the VHT. The draft roadmap proposes the creation of a repository of patient-specific health data, physics-based and data-driven predictive models and a simulation platform, which can enable a fully personalised medicine to support clinical decision making and ISTs. In December 2023, the EC launched the European Virtual Human Twins Initiative.^106^ This is funding research projects and promoting the creation of infrastructures to enable the development and application of computational medicine technologies in the EU healthcare environment. In particular, by aiming at the practical implementation of the needs identified by the EDITH roadmap, the VHT initiative is working towards the creation of a shared European repository of health data and modelling resources, an accessible platform for model development, simulation and computing facilities, and a regulatory, legal and ethical framework for the use of the VHT technologies. Despite the existence of numerous VHT solutions created by academia and industry, the EU's VHT ecosystem remains disjointed and fragmented. By fostering the inclusion and collaboration of diverse stakeholders, promoting the interoperability of health data and in silico models, and accelerating research and technological advancements through supercomputing capacities and AI, the VHT initiative promises to unify the fragmented ecosystem and to overcome the barriers currently preventing the real-world use of computational medicine in healthcare.

## Conclusion

In the coming years, computational medicine will have a growing impact on healthcare, introducing innovative technologies at numerous stages of the healthcare pathway, from disease prevention to diagnostics and treatment. In silico solutions have the potential to bring benefits to all the stakeholders in the healthcare ecosystem: patients can have faster and more accurate diagnosis and better and personalised treatments; clinicians can deploy reliable tools to support the treatment choice; companies can use innovative tools to design and test new products, reducing the time and cost to market. Ultimately, society in general will benefit, with a reduction in the costs of disease management and mitigation of some of the ethical concerns associated with preclinical research and clinical trials.

With a focus on EU legislation and initiatives, we have proposed and applied the concept of ‘computational model lifecycle’ to illustrate three main stages of development of in silico solutions and their translational challenges. As expected with new disruptive technologies, the innovations are outpacing the regulations. This points to the need for an agile regulatory framework which is able to facilitate the technology translation in healthcare and to guarantee the reliability, safety and efficacy of in silico tools.

Health data are the foundation for building in silico models: researchers need to have access to quality data and, especially in the case of data-driven approaches, to large datasets. The EU GDPR and the more recent Data Governance Act regulate important aspects, such as data privacy, anonymization and secondary use. The implementation of the EHDS promises to leverage the value of health data, providing a common platform containing quality and standardised data for primary and secondary use, not only in research and innovation, but also in policy-making and regulatory decision making.

In the EU, the VHT Initiative demonstrates a commitment to strengthen the development of computational medicine technology and in promote its integration into the healthcare ecosystem. However, there are regulatory gaps, such as the official recognition of in silico evidence in the safety and efficacy assessment of medical devices. There are also standardisation gaps, such as adherence to best practices for developing quality-assured physics-based and data-driven models. At present there is no well-established and harmonised approach for developing and enforcing model credibility assessment standards in the EU, even though the EMA and Member State authorities can play a decisive role on a case-by-case basis. The definition of clear guidelines on good simulation practices and credibility assessments of in silico models, similar to the FDA's ASME VV40-2018 standard, would facilitate commercialisation and clinical use, both for sponsors submitting their in silico solutions to regulatory agencies, and for evaluators. A possible approach could be the inclusion of the ASME VV40-2018 framework within EU guidelines, which would also establish a common framework with the U.S. market. However, the scope of the ASME standard is limited to physics-based models. The EU therefore has an opportunity to develop a guideline combining both mechanistic and data-driven modelling approaches, building on the advanced regulatory framework for AI applications.

Some emerging trends and technological advancements in biomedical and AI research promise to enhance the adoption of in silico medicine. The concept of Internet of Medical Things defines the network of medical devices, sensors, wearable devices and software applications enabling the collection, analysis and exchange of medical data.^107^ The growing diffusion of wearables and implantable sensors, together with the digitalisation of healthcare, will contribute to the generation of vast amount of data which can be used to develop in silico medicine models, according to regulations on secondary uses of health data.^83,86,102^ Research on AI for healthcare is focused on technical aspects such as multimodal data integration, to improve model performance by integrating multiple sources of information, and model explainability, to increase the trust in model predictions.^83^ Other research efforts focus on making mechanistic models more scalable, by exploring the use of ML approaches to speed-up the generation of desired outputs.^82,102,108,109^

The future of computational medicine holds great promise, but to unlock its full potential in the EU, there is a need to address a series of technical, regulatory and standardisation gaps. Synergies between researchers, industry stakeholders, healthcare professionals, policy-makers and regulatory bodies are fundamental to accelerate the integration of computational medicine in healthcare. An early collaboration between researchers, clinicians and industry favours the identification of healthcare needs to address with in silico solutions, and the building of trust in the process of model development and application. It would also foster a translational research approach, promoting the advancement of research projects through different stages of the computational model lifecycle. With the same aim, the available standards and guidelines should be adopted early in the process of model development, to facilitate eventual use in pre-clinical and clinical applications. Policy-makers can contribute by developing and implementing supportive initiatives, providing funding opportunities and encouraging data sharing and collaborative research, similar to what is planned for the VHT Initiative. Researchers should embrace these initiatives and be open to collaborative and shared efforts, to facilitate model adoption and further development. Finally, the integration of experts in computational modelling in regulatory bodies would enable the development and implementation of suitable and effective regulatory frameworks and guidelines, taking into account crucial aspects such as data and model credibility, interoperability and transparency. By establishing clear guidelines and frameworks, the EU can facilitate the development and adoption of innovative computational models, ultimately driving improvements in patient outcomes, healthcare efficiency, and societal well-being.
